# Regulation of mitochondrial dynamics in cardiomyocytes: implications for cardiac health and disease

**DOI:** 10.3389/fcell.2025.1652683

**Published:** 2025-09-10

**Authors:** Tengxu Zhang, Ziwei Li, Ying Xu, Chaoqun Xu, Hao Wang, Tao Rui

**Affiliations:** Division of Cardiology, Department of Medicine, The Affiliated People’s Hospital of Jiangsu University, Zhenjiang, Jiangsu, China

**Keywords:** mitochondrial dynamics, DRP1, Mfn1, MFN2, OPA1, sepsis, diabetic cardiomyopathy, I/R injury

## Abstract

Mitochondrial dynamics, involving fission and fusion, are vital for maintaining mitochondrial quality, shape, and function in heart cells. This review explores how key regulators—Dynamin-related protein 1 (Drp1), mitofusins 1 and 2 (Mfn1/2), and Optic Atrophy 1 (OPA1)—control these processes in the heart. Drp1 facilitates fission, while Mfn1/2 and OPA1 mediate outer and inner membrane fusion. Their activities are finely tuned by modifications, gene regulation, and stress pathways. Disruptions in these dynamics can impair functions like energy production, calcium balance, ROS management, and mitophagy, contributing to heart diseases. Abnormal fission and fusion are also linked to conditions such as sepsis, ischemia/reperfusion injury, and diabetic cardiomyopathy. This review aims to offer a thorough analysis of recent advancements in the understanding of dysregulated mitochondrial dynamics and their contribution to cardiac pathology. Additionally, it evaluates emerging therapeutic strategies that target the balance between mitochondrial division and fusion. We posit that precise modulation of the activities of Drp1, Mfn1/2, and OPA1 presents significant potential for the treatment of cardiac diseases. However, achieving tissue specificity and temporal control remains a critical challenge for clinical translation.

## 1 Introduction

Mitochondria are involved in many key cellular functions, such as cellular respiration, cell differentiation, apoptosis (Song and Dorn, 2015), and calcium signaling (Garbincius and Elrod, 2022). The organelle’s shape is most closely related to its primary function of oxidative phosphorylation. Mitochondria have an outer and inner membrane. The outer mitochondrial membrane (OMM) separates mitochondrial contents from the cytosol. The inner mitochondrial membrane (IMM) is convoluted into cristae and contains proteins for various mitochondrial functions (Gao and Hu, 2021). Between the two membranes is the intermembrane space (IMS), which contains the proton gradient needed for ATP synthesis (Horvath and Daum, 2013). Inside the IMM is the mitochondrial matrix, which contains other proteins and mitochondrial DNA (mtDNA) (Yan et al., 2019).

The OMM is important because it compartmentalizes mitochondrial contents to protect the cytosol from biochemical reactions occurring within the organelle. The OMM also regulates mitochondrial morphology based on cellular needs and mediates removal of damaged mitochondria (Chu et al., 2013). The IMM is also important as it is the site for many mitochondrial functions, including the electron transport chain (ETC), phospholipid metabolism, and regulation of apoptotic signaling (Westermann, 2010). Dysfunction of these mitochondrial processes is associated with many diseases, including cancer, diabetes, and neurodegenerative diseases (Rainbolt et al., 2015).

Mitochondria are particularly important in myocardial cells, including cardiac myocytes (CM) and cardiac fibroblasts (CF), as the heart is an organ that consumes a lot of energy. In fact, CMs exhibit the highest mitochondrial density among cell types, with mitochondria occupying approximately 40% of the cell volume, and consequently possess the highest respiratory capacity (Da Dalt et al., 2023). Mitochondria in adult cardiac muscle fibers are highly organized and divided into three subpopulations: those compacted between contractile filaments in lanes parallel to the long axis (interfibrillar), those found adjacent to sarcolemma (subsarcolemmal) (Chen et al., 2012), and those found around the nucleus (perinuclear) (Sanchis-Gomar et al., 2016). The subpopulations demonstrate unique functional specializations. Interfibrillar mitochondria (IFM) predominantly generate ATP to directly energize the contractile apparatus (Vendelin et al., 2005). Subsarcolemmal mitochondria (SSM) are strategically located to facilitate ion transport across the sarcolemma and may also play a role in cellular signaling (Dorn and Kitsis, 2015). Perinuclear mitochondria (PNM) are hypothesized to supply energy for nuclear processes and potentially contribute to the regulation of gene expression associated with mitochondrial biogenesis (Zhu et al., 2022). These mitochondria are essential for the proper functioning of the CM and CF, and by extension, of the heart itself.

Mitochondria play a pivotal role in cardiac energy metabolism and cellular survival, making their dynamic remodeling through fission and fusion processes crucial for maintaining myocardial health. Future research should focus on elucidating the differential contributions of mitochondrial subpopulations to the progression of cardiac diseases.

## 2 Mitochondrial dynamics and myocardial function: theoretical foundations

### 2.1 Evolution of mitochondrial dynamics concepts in cardiac cells

The dynamics of mitochondria within cardiac myocytes have been the subject of extensive research, leading to a substantial evolution in conceptual understanding over time. Foundational studies, including those that have quantitatively assessed the spatial distribution and motility of mitochondria in adult rat cardiomyocytes and non-beating HL-1 cells, were particularly influential (Beraud et al., 2009).

Recent research has challenged the traditional notion that mitochondria in adult cardiomyocytes are static entities. For instance, certain studies have reexamined mitochondrial dynamics and proposed that the assertion of high mitochondrial dynamics may not be applicable to all cell types (Dorn, 2019). Historically, investigations involving *in vivo* experimental manipulation of mitochondrial fusion and fission genes in cardiomyocytes provided limited evidence for mitochondrial dynamics (Dorn, 2018). However, these studies have illuminated the role of dynamic factors in regulating mitochondrial mass. This indicates that targeting mitochondrial dynamics proteins within the cardiac system could potentially uncover novel functions of these factors in biological pathways.

### 2.2 Mitochondrial dynamics

**FIGURE 1:**
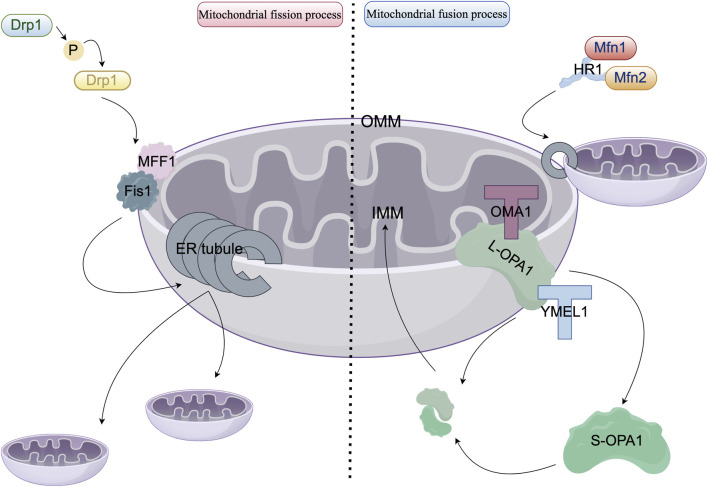
Molecular mechanisms governing mitochondrial fission and fusion. In the left panel, the cytoplasmic dynamin-related protein 1 (Drp1, depicted in blue) phosphorylates Ser616 (highlighted in yellow) via anchoring proteins, specifically Fis1 (shown in gray) and MFF1 (illustrated in pink), at sites of contact with the endoplasmic reticulum (ER, represented as gray tubules). Drp1 undergoes oligomerization into GTP-hydrolyzing helical polymers that facilitate membrane constriction and drive mitochondrial division. In the right panel, mitochondrial fusion proteins Mfn1 (colored red) and Mfn2 (colored orange) form antiparallel dimers through interactions with the HR1 domain. GTP hydrolysis induces a conformational change that enables the formation of the outer mitochondrial membrane (OMM) fusion pore. Optic atrophy 1 (OPA1, shown in dark green) is localized within the inner mitochondrial membrane (IMM) and produces a soluble short form (S-OPA1, also in dark green) via proteolytic cleavage by OMA1 (at the S1 site, marked with a red T-shape) and YME1L (at the S2 site, marked with a blue T-shape). Both L-OPA1 and S-OPA1 assemble into GTP-dependent complexes that facilitate IMM fusion and cristae reorganization.

The prevailing perspective posits that mitochondria are not static organelles; rather, they demonstrate dynamic behavior, frequently forming interconnected networks with filamentous structures that, in certain cell types, facilitate the transmission of signals between mitochondria in wave-like patterns (Bhandari et al., 2015). Within cells, these networks often undergo remodeling through mitochondrial division and fusion processes (Baker et al., 2014). Mitochondrial dynamics, encompassing fission, fusion, and transport processes, are essential for mitochondrial quality control (Cahill et al., 2015; Heidenreich et al., 2022). Figure 1 depicts the detailed process of mitochondrial fusion and fission, highlighting the mechanisms of action of key molecular components. HR1 (Heptad Repeat Domain 1) represents a crucial functional domain within the mitochondrial outer membrane fusion proteins, Mitofusin 1 and 2 (Mfn1/Mfn2). This domain encompasses a conserved amphipathic helix, spanning residues 393 to 410, which integrates into the membrane, specifically targeting regions characterized by packing defects. This integration destabilizes the lipid bilayer, facilitating the fusion of the outer mitochondrial membrane independently of HR2-mediated docking. Such fusion is vital for the maintenance of the mitochondrial network, as the absence of HR1 significantly impairs cellular fusion processes (Daste et al., 2018).

Mitochondrial dynamics play crucial roles in numerous cellular functions. These include the segregation of mtDNA and proteins during mitosis, mitophagy, apoptosis, and cell differentiation. Additionally, dynamics are involved in calcium regulation (Gonzalez et al., 2014), distribution of mitochondria-derived metabolites, oxygen sensing, maintenance of mitochondrial morphology and function, and modulation of mtDNA replication (Archer, 2013). These dynamic processes facilitate the positive cellular regulation of mitochondria, and any imbalance in mitochondrial dynamics can result in pathological conditions such as inflammation or heart failure.

Mitochondrial fission and fusion are orchestrated by four GTPases belonging to the mitochondrial dynein family: Mfn1 and Mfn2 facilitate fusion of the mitochondrial OMM, while OPA1 is responsible for fusion of the mitochondrial IMM. Drp1 governs mitochondrial fission. The regulation of these proteins is multifaceted, encompassing transcriptional control, alternative splicing, and post-translational modifications (Sharp and Archer, 2015). Additionally, these proteins are responsive to various physiological stimuli. For instance, conditions such as starvation and stress suppress division and encourage the formation of mitochondrial networks through fusion, whereas mitochondrial damage and depolarization inhibit fusion. Short-term stress conditions enhance fusion, whereas prolonged stress leads to mitochondrial fragmentation (Redpath et al., 2013; Tao et al., 2018). Moreover, the processes of fission and fusion are influenced by the activity of the endoplasmic reticulum (ER) and the PINK1-Parkin mitophagy pathway (Liu et al., 2023). In murine models, the genetic ablation of factors involved in cellular division and fusion results in embryonic lethality, highlighting the essential role of these processes in ensuring survival (Adebayo et al., 2021). Conversely, in humans, genetic syndromes arising from mutations in these genes are nonfatal and primarily impact specific regions of the nervous system (Malakauskaite et al., 2024).

While mitochondrial dynamics are well-characterized in non-cardiac myocytes, the spatiotemporal regulation of these processes in actively contracting cardiac myocytes necessitates further investigation. Critical areas of uncertainty include the mechanisms by which mechanical stress from contraction influences the localization of Drp1 and Mfn2, as well as potential variations in cristae dynamics across distinct subsets of cardiac mitochondria.

## 3 Important molecules affecting mitochondrial dynamics

Mitochondrial dynamics involve numerous critical molecules, each responsible for regulating distinct processes. This chapter primarily elucidates the specific roles, activation, and regulatory mechanisms of four key molecules: Drp1, Mitofusin-1, Mitofusin-2, and OPA1. Table 1 provides a summary of the functions and regulatory pathways of these core kinetic proteins. Table 2 provides a summary of molecular regulatory mechanisms of key proteins in mitochondrial dynamics.

**TABLE 1:** Summary of functions and regulation of core mitochondrial dynamics proteins.

Molecule	Function	Mechanisms of regulation	Cardiac phenotype in deficiency/Knockout
Drp1	Mediates fission	Phosphorylation (S616: activation)	Mitochondrial elongation (impaired fission) Impaired mitophagy Heart failure
		Phosphorylation (S637: inhibition)	
Mfn2	Mediates outer membrane fusion	Transcriptional upregulation (e.g., by PGC1α)	Impaired respiration Increased ROS Cardiomyopathy
	Regulates ER-mitochondria tethering (MAMs)	Ubiquitin-mediated degradation (e.g., by Parkin)	
OPA1	Mediates inner membrane fusion	Proteolytic cleavage (OMA1/YME1L)	Cristae disorganization Reduced ATP production Increased susceptibility to I/R injury
	Maintains cristae structure	Balance of L- and S-OPA1 isoforms	

**TABLE 2:** Molecular regulatory mechanisms of key mitochondrial dynamics proteins.

Protein	Regulatory mechanism	Specific regulatory events
Drp1	Post-translational modifications	Phosphorylation (e.g., S616: activation; S637: inhibition), ubiquitination, SUMOylation, S-nitrosylation, acetylation
Mfn1/2	Transcriptional regulation	Upregulated by PGC1α
	Post-translational modification (ubiquitination and degradation)	Ubiquitin-mediated degradation (e.g., by Parkin) (PINK1-Parkin pathway); regulated by BAT3 (under specific conditions)
OPA1	Proteolytic processing	Cleaved by proteases OMA1 (at S1 site) and YME1L (at S2 site); cleavage generates soluble S-OPA1
	Alternative splicing	Generates multiple isoforms: L-OPA1 (a, b) and S-OPA1 (c, d, e)

### 3.1 Drp1, role of Drp1 in mitochondrial dynamics, how Drp1 activity is regulated

Drp1 is a predominantly cytosolic member of the dynamin family of GTPases, playing a crucial role in mitochondrial fission (Tao et al., 2018; Kraus et al., 2021). Upon activation, Drp1 is recruited to the OMM, where it oligomerizes, hydrolyzes GTP, and forms spiral structures around mitochondria, thereby constricting the membranes to initiate mitochondrial fission (Tilokani et al., 2018). This recruitment and constriction process can be facilitated by forces generated by the actin cytoskeleton (Mears and Ramachandran, 2022). This assembly process involves interactions with OMM-spanning proteins, often occurring at contact sites between mitochondria and the ER (Wai et al., 2015). At the endoplasmic reticulum-mitochondria contact sites (MERCs), actin polymerization associated with the endoplasmic reticulum, which is nucleated by proteins such as the ARP2/3 complex and its regulators can produce forces that facilitate mitochondrial constriction preceding the action of Drp1 or work in conjunction with Drp1 during the fission process (Gatti et al., 2025). The fission assembly is further facilitated by non-GTPase proteins, including mitochondrial fission protein 1 (Fis1), mitochondrial fission factor 1 (MFF1), Mid49, and Mid51. Additionally, ER tubules encircle mitochondria prior to fission (Seo et al., 2019).

Drp1 activity is modulated by various post-translational modifications, such as phosphorylation (Gao et al., 2022), ubiquitination (Sulkshane et al., 2021), sumoylation (Jin et al., 2021), N-nitrosylation, and acetylation (Hu Q. et al., 2020). Phosphorylation plays a dual role: it can either activate or inhibit Drp1. Specifically, phosphorylation at Drp1-serine 616 activates the protein and promotes its translocation to the OMM, while phosphorylation at Drp1-serine 637 exerts an inhibitory effect (Kim et al., 2019). The dynamic phosphorylation and dephosphorylation of these sites have implications in the development of pathological conditions.

Through a series of overexpression and knockout studies, Drp1 has been implicated in mitochondrial fragmentation, mitophagy, and apoptosis. In *drosophila*, Drp1 overexpression induces mitochondrial fragmentation without affecting cardiac function (Jenkins et al., 2024). While some studies suggest that increased mitochondrial fission can confer protection against apoptosis. For instance, mitochondrial fission promotes anti-apoptotic mechanisms by activating protective signaling pathways, such as the Reperfusion Injury Salvage Kinase (RISK) pathway and the cAMP response element-binding protein (CREB) pathway (Rosdah et al., 2016). Simultaneously, mitochondrial fission plays a crucial role in preserving mitochondrial function and homeostasis. Proper and moderate mitochondrial division is essential for maintaining the appropriate number, distribution, and functionality of mitochondria, thereby ensuring the stability of the internal environment, including energy supply and redox balance. When the regulatory mechanisms governing mitochondrial division operate effectively, they can prevent detrimental outcomes such as excessive cellular apoptosis and safeguard the survival of cardiomyocytes (Long et al., 2020), others indicate that Drp1 is crucial for apoptosis, a process that involves mitochondrial fragmentation (Ansari et al., 2022). Drp1 has been demonstrated to facilitate bax oligomerization and cytochrome c release (Pena-Blanco and Garcia-Saez, 2018). Consequently, there have been efforts to inhibit Drp1 to mitigate I/R injury (Zeng et al., 2022). Drp1, along with other regulators of mitochondrial morphology, is encoded by the nuclear genome. Consequently, alterations in mitochondrial form and function are likely to exhibit tissue-specific characteristics. Research has demonstrated that Drp1 is crucial for the normal development and functioning of the heart and brain. However, its short-term inhibition in the context of ischemia-reperfusion (IR) injury has been shown to confer cardioprotective and neuroprotective effects, suggesting its potential as a therapeutic target in cardiac arrest scenarios (Sharp, 2015). However, it is hypothesized that a basal level of Drp1 activity may be necessary for maintaining mitophagy and ATP synthesis.

Furthermore, it is noteworthy that mitochondrial division process 1 (MTFP1), also referred to as MTP18, is a protein located in the mitochondrial inner membrane (IMM) that has emerged as a crucial regulator of mitochondrial division. It operates downstream of, and in conjunction with, Drp1. The expression of MTFP1 is transcriptionally regulated by the PI3K/Akt/mTOR signaling pathway, thereby linking cell growth and metabolic signals directly to the mitochondrial fission machinery (Morita et al., 2017; Tabara et al., 2024). MTFP1 facilitates mitochondrial division by promoting the recruitment and assembly of Drp1 oligomers at the division site. Recent research has uncovered new roles for MTFP1 in regulating bioenergetic efficiency and sensitivity to cell death, as well as highlighting its significance in preventing pathogenic cardiac remodeling (Donnarumma et al., 2022).

The loss of Drp1 in cardiac tissue is associated with lethality. Specifically, the knockout of Drp1 in the heart impairs mitochondrial quality control and mitophagy, resulting in enlarged and abnormal mitochondria with reduced numbers (Tong et al., 2020). Previous studies have shown that cardiomyocyte-specific knockdown of Drp1 in mice leads to impaired mitochondrial quality control and mitophagy. This damage leads to the accumulation of enlarged and abnormal mitochondria and ultimately contributes to programmed cardiomyocyte necrosis through mPTP opening, myocardial fibrosis and fatal heart failure, but whether DRP1 is directly involved in the execution of mitophagy is still controversial (Tong et al., 2023). This condition leads to programmed cardiomyocyte necrosis, myocardial fibrosis, and ultimately, fatal heart failure. The heart failure observed may be attributed to diminished mitophagy and the accumulation of damaged mitochondria. This reduction in mitophagy is characterized by an initial acceleration of early mitophagic processes, followed by impaired mitochondrial import into lysosomes, leading to fission without proper mitophagic degradation and a general loss of mitochondria (Lin et al., 2022). Necrosis is eventually induced through the activation of the mitochondrial permeability transition pore (mPTP) (Duan et al., 2021). Conversely, other studies have shown that inhibition of Drp1 reduces cytochrome c release, thereby preventing cell death.

Although Drp1 is recognized as the primary GTPase governing mitochondrial fission, it is crucial to acknowledge the identification of alternative, Drp1-independent fission pathways under certain conditions. For instance, mechanisms of division that are independent of Drp1 have been identified in contexts such as bacterial infection-induced division, the formation of mitochondrial-derived vesicles (MDVs), and division during autophagy. Furthermore, the review underscores the significance of these DrP1-independent division mechanisms (Pagliuso et al., 2018). Nonetheless, Drp1 remains the central and predominant regulator of mitochondrial fission in the majority of physiological and pathological contexts.

### 3.2 Mitofusin-1/2 (Mfn1/2), role of Mfn1/2 in mitochondrial dynamics, how Mfn1/2 activity is regulated

Mitofusin-1 (Mfn1) is a dynamin-like GTPase that, along with Mitofusin-2 (Mfn2), consistently spans the outer mitochondrial membrane (OMM) via two transmembrane segments, with their N-terminal GTPase domains oriented towards the cytoplasm (Tabara et al., 2025). The mitofusins play a crucial role in the fusion of the OMM, operating as either Mfn1/Mfn2 heterodimers, Mfn2/Mfn2 homodimers, or Mfn1/Mfn1 homodimers (Hall et al., 2014). Beyond their role in OMM fusion, mitofusins also facilitate the interaction between cardiac mitochondria and the sarcoplasmic reticulum (SR), thereby enhancing calcium signaling as part of the endoplasmic reticulum stress response. Additionally, the expression levels of both mitofusins are elevated in human heart failure (Chen et al., 2012).

Both mitofusins are transcriptionally regulated by peroxisome proliferator-activated receptor gamma coactivator1-α (PGC1α) (Dorn et al., 2015), which is also implicated in mitochondrial biogenesis. In addition to transcriptional regulation, mitofusins may undergo post-translational ubiquitin-mediated degradation, potentially regulated by human leukocyte antigen B-associated transcript 3 (BAT3). BAT3 interacts with Mfn2 and is essential for the degradation of mitofusins under conditions of Drp1 depletion, possibly by facilitating their recruitment to the proteasome. Furthermore, the ubiquitin-mediated degradation of mitofusins may also be regulated by Parkin, an E3 ubiquitin ligase, thereby linking mitofusins to the PINK1-Parkin mitophagy pathway (Deng et al., 2024).

The two mitofusins appear to be functionally redundant in terms of mitochondrial fusion, as evidenced by the minimal alterations in mitochondrial morphology observed in MEF cells when one mitofusin is absent (Hu et al., 2021). In contrast, the simultaneous absence of both mitofusins results in significantly shorter and partially depolarized mitochondria. Despite their redundancy, Mfn1 and Mfn2 exhibit distinct functional characteristics. Specifically, Mfn1 demonstrates more efficient GTPase activity and plays a role in remodeling the OMM (Papanicolaou et al., 2012). Consequently, the loss of either Mfn1 or Mfn2 can only be partially compensated by the presence of the other protein. Notably, the deficiency of one mitofusin leads to the upregulation of the other.

Both mitofusins are essential for development, as the absence of either mitofusin in the germline results in lethality, likely attributable to defects in placental development (Dorn, 2015). While selective ablation of Mfn1 is generally well-tolerated, the loss of Mfn2 leads to functional impairments (Rovira-Llopis et al., 2017; Zhang et al., 2022). CM-specific knockout of either mitofusin confers protection against I/R injury in cardiac muscle; however, the knockout of both mitofusins results in rapidly fatal cardiac failure. In adult mouse hearts, CM-specific ablation of both mitofusins results in an increased number of small mitochondria, impaired respiration, elevated ROS levels, and eccentric cardiomyopathy due to impaired mitophagy, despite the absence of CM loss. In *Drosophila*, CM-specific silencing of MARF, the *Drosophila* equivalent of mitofusin, leads to the formation of small, depolarized mitochondria in hypercontractile heart tubes and increased ROS production (Jiang et al., 2022). Mice deficient in mitofusins in skeletal muscle develop a lethal mitochondrial myopathy, likely due to a significant reduction in mtDNA levels and an increase in mtDNA point mutations and deletions (El-Hattab et al., 2017).

### 3.3 OPA1, role of OPA1 in mitochondrial dynamics, how OPA1 activity is regulated

OPA1 is a dynamin-like GTPase located in the IMS and anchored within the IMM (Noone et al., 2022). OPA1 is responsible for IMM fusion and is a key regulator of the fusion/fission balance, as well as other processes. Its activity is regulated by proteolytic processing (Yang et al., 2015).

The OPA1 protein contains major cleavage sites, S1 and S2, cleaved by the proteases OMA1 and YME1L, respectively (Anand et al., 2014). These sites will be examined in greater detail in the subsequent sections. Cleavage at these sites generates soluble forms of OPA1 (S-OPA1) that lack the transmembrane domain. Additionally, OPA1 can undergo alternative splicing, resulting in isoforms that exhibit differential expression across various tissues. There are five known isoforms of OPA1: a, b, c, d, and e. Isoforms a and b are classified as long forms (L-OPA1), whereas isoforms c, d, and e are considered short forms (S-OPA1) (Rainbolt et al., 2016). Some studies suggest that constitutive OPA1 processing is essential for maintaining normal mitochondrial morphology, with L-OPA1 and S-OPA1 hypothesized to collaborate in facilitating mitochondrial fusion and assembling complexes that preserve cristae structure (Fry et al., 2024). Conversely, other research indicates that L-OPA1 alone is sufficient for fusion, while S-OPA1 is associated with mitochondrial fission. “Notably, GTPase-inactive S-OPA1 has been observed to partially localize to mitochondria-associated ER membranes (MAMs) (Fogo et al., 2024).

OPA1 has multiple functions beyond mediating mitochondrial fusion. It plays a crucial role in maintaining the proper architecture of the IMM cristae, as evidenced by several studies (Zhang et al., 2014). The morphology of cristae is vital for tissue homeostasis and the regulation of cell death. Both long and short forms of OPA1 (L-OPA1 and S-OPA1) form complexes that are essential for the maintenance of cristae structure. OPA1 facilitates the formation of tight cristae junctions, thereby modulating apoptotic cristae remodeling and protecting cells from apoptosis. The stabilization of cristae by OPA1 enhances mitochondrial respiratory efficiency and reduces mitochondrial dysfunction, cytochrome c release, and ROS production (Liang et al., 2024). Moreover, OPA1 is involved in the regulation of oxidative phosphorylation and the stabilization of ETC complexes, which leads to an increase in respiratory capacity (Su et al., 2023b). Furthermore, OPA1 regulates mtDNA. Some studies suggest that OPA1 may reduce mtDNA copy number by inhibiting replication or promoting the accumulation of deletions. By promoting fusion, OPA1 contributes to mitochondrial elongation and network formation (He et al., 2022).

While OPA1 does not enhance mitochondrial biogenesis or inhibit autophagy, it is crucial for sustaining mitochondrial functional integrity (Wang et al., 2024). It is known that OPA1 undergoes degradation in depolarized mitochondria. In cases of heart failure (HF), OPA1 levels are reduced, although it remains uncertain whether this reduction is a consequence of HF or a contributing factor (Schwartz et al., 2022). Mutations in OPA1 cause autosomal dominant optic atrophy (DOA), a neurodegenerative disorder primarily affecting the optic nerve. Overexpression of OPA1 has been shown to confer protection against mitochondrial diseases and apoptotic stimuli, and it can induce mitochondrial elongation (Cartes-Saavedra et al., 2023). While some studies suggest that OPA1 overexpression may be toxic in mice, mild overexpression does not appear to impact lifespan. Controlled OPA1 overexpression mitigates injury in highly metabolically active organs. This protection is achieved by reducing cristae remodeling, cytochrome c release, and mitochondrial dysfunction, thereby counteracting ischemic damage. Additionally, it can induce physiological cardiac hypertrophy and potentially reduce body weight, although it may also increase the risk of spontaneous tumorigenesis.

A reduction in OPA1 levels has numerous adverse consequences, attributable to the multifaceted roles of OPA1. This reduction can result in compromised mitochondrial fusion and cristae architecture, heightened vulnerability to apoptosis, respiratory dysfunction, and excessive ROS production (Pernas and Scorrano, 2016). In the absence of OPA1, OMMs can still undergo fusion, but IMMs cannot, leading to metabolic disturbances and preventing mixing of matrix contents between mitochondria (Nyenhuis et al., 2023). In *Drosophila*, OPA1 silencing in cardiac tissue results in cardiac dysfunction, mitochondrial depolarization, and increased ROS production (Dorn et al., 2011). In mice, both constitutive and tissue-specific ablation of OPA1 is embryonically lethal, while heterozygous OPA1 ± mice exhibit late-onset cardiac dysfunction at 1 year of age, characterized by reduced heart size and function. These mice exhibit small, fragmented mitochondria with damaged cristae. Furthermore, mitochondrial density is reduced, and their arrangement between myofilaments is disorganized. These defects culminate in impaired cardiac mitochondrial function and reduced ATP production. Additionally, they exhibit a decreased mtDNA copy number and may eventually develop blindness (Huang et al., 2017). The concomitant increase in ROS renders the myocardium more susceptible to I/R injury. Nonetheless, some studies reported no significant increase in apoptosis or cardiomyocyte loss in these heterozygous OPA1 ± mice.

Drp1, Mfn1/2, and OPA1 constitute a cohesive regulatory network that responds to metabolic stress. Post-translational modifications, including Drp1 phosphorylation and OPA1 cleavage, serve as critical intervention nodes that significantly impact the mitochondrial quality control system. Future research should aim to elucidate the distinct outcomes associated with various intervention nodes, thereby enhancing our understanding of the intricate regulation of mitochondrial quality control.

## 4 Imbalance of mitochondrial fission and fusion in myocardial pathologies

**FIGURE 2:**
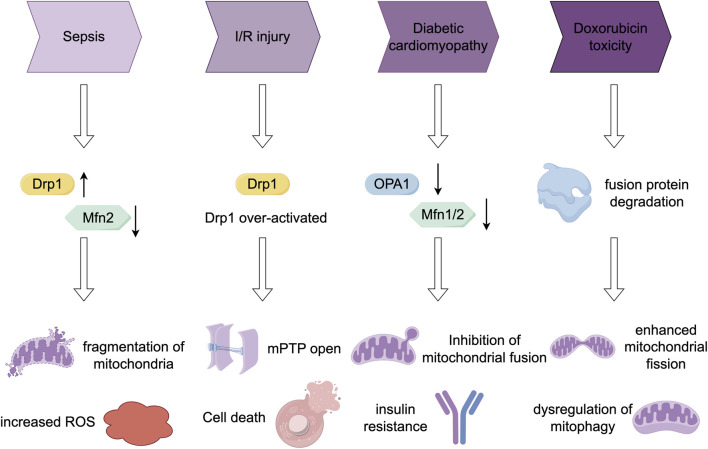
Comparison of mitochondrial dynamics imbalance in four disease models.

In this section, we examine the aberrant regulation of mitochondrial fission and fusion processes in various myocardial pathologies. We elucidate alterations in key molecular regulators across different pathological conditions and the resultant functional consequences. Additionally, we compile intervention strategies suggested by contemporary research. Figure 2 provides a summary of the dynamic changes in protein expression and the effects of interventions in models of these diseases.

### 4.1 Sepsis

Sepsis-associated organ failure is characterized by increased ROS production and mitochondrial dysfunction (Lira Chavez et al., 2023). An imbalance in mitochondrial fission and fusion processes may contribute to the pathophysiology of sepsis. Some sepsis models have demonstrated a reduction in Mfn2 mRNA levels and an elevation in Drp1 mRNA levels, which are associated with mitochondrial fragmentation (Ying et al., 2017; Wu et al., 2023). Recent research on MFN2 splice variants, specifically ERMIT2 and ERMIN2, has elucidated their pivotal function in sustaining ER-mitochondria tethering and calcium transfer (Naon et al., 2023). These findings may offer novel insights into the mitochondrial dysfunction associated with sepsis. The efficacy of treatments in restoring mitochondrial fusion varies across different sepsis models, although recovery has been observed in lipopolysaccharide (LPS)-treated animals. The compound mdivi-1, a Drp1 inhibitor, has been shown to preserve mitochondrial function and mitigate apoptosis in a caecal ligation and puncture model of sepsis (Deng et al., 2020). The study conducted by Bordt et al. (2017) offers a significant revision to the traditional understanding, demonstrating that Mdivi-1 is not a specific inhibitor of DRP1. Instead, it primarily exerts its effects through the reversible inhibition of mitochondrial complex I, a mechanism that operates independently of Drp1. This compound’s capacity to modulate reactive oxygen species (ROS), particularly by inhibiting reverse electron transport (RET) ROS, may elucidate its protective effects observed in various disease models. Furthermore, RCAN1 deficiency has been shown to aggravate sepsis-induced cardiac remodelling and dysfunction by accelerating pathological mitochondrial fission (Zhuang et al., 2022).

### 4.2 Myocardial ischemia/reperfusion injury

Numerous studies have established a correlation between I/R injury and dysregulation of mitochondrial fission/fusion dynamics (Bai et al., 2023). Specifically, mitochondria in ischemic cells undergo Drp1-dependent fission. However, studies have shown that the TBC1D15-Drp1 interaction, which mediates mitochondrial homeostasis, confers cardio protection against myocardial I/R injury (Sun et al., 2022). Drp1-mediated fission promotes mitochondrial ROS production, elevates cytosolic calcium levels, and impairs diastolic relaxation (Tao et al., 2018; Du et al., 2022). Consequently, inhibiting mitochondrial fission represents a protective strategy against I/R injury. Inhibiting Drp1, either by overexpressing a dominant-negative form or using mdivi-1, has been shown to enhance cell survival and delay mitochondrial permeability transition pore (mPTP) opening following simulated I/R injury in cardiomyocytes (Ong et al., 2010). Additionally, the Drp1 inhibitor P110 has demonstrated efficacy in reducing brain I/R injury when administered at reperfusion (Liu et al., 2022). Separately, inhibition of Drp1 (and Fis1) has been shown to decrease right ventricular dysfunction in pulmonary hypertension models of I/R (Tian et al., 2017). However, it is important to consider that a low level of chronic Drp1 activation may be necessary for processes such as mitophagy and ATP synthesis (Schmitt et al., 2018). Furthermore, additional Drp1 inhibitors, such as Drpitor1a, have demonstrated potential cardioprotective effects. Drpitor1a has been shown to decrease infarct size and enhance cardiac function in a mouse model of myocardial ischemia-reperfusion (I/R) injury by directly inhibiting the GTPase activity of Drp1 and mitigating mitochondrial over division (Piao et al., 2024). Conversely, S1QELs, which are specific inhibitors of mitochondrial complex I superoxide production, have exhibited significant myocardial protection in an I/R injury model by specifically inhibiting Q superoxide production at the complex I site (Watson et al., 2019).

Ischemic preconditioning (IPC)—a phenomenon characterized by brief ischemic episodes that confer protection against subsequent prolonged ischemia-reperfusion (I/R) injury—plays a pivotal role in modulating mitochondrial dynamics to achieve cardio protection. IPC effectively enhances fusion processes dependent on OPA1 and MFN2, thereby reducing infarct size and preserving mitochondrial function (Leurcharusmee et al., 2022). Despite the challenges associated with the clinical translation of IPC, its underlying mechanisms highlight the therapeutic potential of targeting mitochondrial dynamics in the context of I/R injury.

The immunoproteasome subunit β2i ameliorates myocardial ischemia/reperfusion injury by regulating mitochondrial fusion (Su et al., 2023a). Overexpression of OPA1 confers protection against ischemic damage, whereas heterozygous OPA1 ± mice exhibit increased ROS, rendering the myocardium more susceptible to ROS-induced I/R injury. Genetic inhibition of the OPA1-mediated cristae remodeling pathway also offers protection against ischemic damage in both cardiac and cerebral tissues (Varanita et al., 2015).

### 4.3 Diabetic myocardial dysfunction

Mitochondrial dysfunction has been implicated in the pathogenesis of type 1 diabetes, type 2 diabetes, and obesity, with diabetes serving as a risk factor for HF (Belosludtsev et al., 2020). In type 2 diabetes, cardiomyocytes exhibit damaged and depolarized mitochondria, characterized by impaired fatty acid oxidation, reduced mitochondrial content, decreased oxidative phosphorylation capacity, and increased ROS production (Pinti et al., 2019). Various studies have demonstrated that individuals with diabetes exhibit reduced expression of mitochondrial fusion proteins, such as Mfn2, Mfn1, and OPA1 (Hu S. et al., 2020). As evidenced by studies (Naon et al., 2023), a decrease in MFN2 expression results in compromised lipid transfer between the endoplasmic reticulum and mitochondria, subsequently inducing endoplasmic reticulum stress and inflammation. These mechanisms may play a contributory role in the pathogenesis of diabetic cardiomyopathy. Cellular models of diabetes reveal mitochondrial fragmentation and diminished fusion (Chang et al., 2022). In cardiomyocytes, exposure to high glucose levels results in the formation of short and small mitochondria, whereas in pancreatic β-cells, a reduction in OPA1 levels is observed prior to the onset of diabetes (Vasquez-Trincado et al., 2016). Mouse models of diabetes exhibit mitochondrial fragmentation, potentially linked to increased OPA1 cleavage. Furthermore, endothelial cells in diabetic models display elevated Drp1 levels, decreased OPA1, and consequently, enhanced mitochondrial fission.

Mitochondrial dynamics and ROS production in the diabetic heart have a bidirectional relationship. While diabetes-induced increases in mitochondrial ROS production influence mitochondrial dynamics, enhanced mitochondrial fission may also contribute to elevated ROS production in the diabetic heart (Atici et al., 2023). Downregulation of Mfn2 exacerbates diabetic cardiomyopathy (Hu et al., 2019), and recent studies have shown that brain natriuretic peptide (BNP) protects against diabetic cardiomyopathy by promoting OPA1-mediated mitochondrial fusion through activation of the PKG-STAT3 pathway (Chang et al., 2023).

The inhibition of mitochondrial fission has been demonstrated to enhance muscle insulin signaling and systemic insulin sensitivity, while also reducing ROS production and cell death (Wang et al., 2023). Elevating OPA1 levels can prevent high glucose-induced mitochondrial fragmentation and dysfunction (Liu et al., 2021). Nonetheless, mitochondrial fragmentation might represent an adaptive response to high glucose under specific cellular contexts. Insulin treatment of cardiomyocytes or skeletal muscle cells can elevate OPA1 levels and promote mitochondrial fusion. In cells deficient in OPA1 and Mfn2, the effects of insulin are compromised.

### 4.4 Doxorubicin-induced myocardial apoptosis

Doxorubicin (DOX), an anthracycline antibiotic, is extensively used in the treatment of various malignancies. However, its clinical application is limited by dose-dependent cardiotoxicity, which arises partly from mitochondrial damage, particularly given the heart’s high mitochondrial density (Kong et al., 2022). DOX administration causes mitochondrial dysfunction, characterized by impaired oxidative phosphorylation, reduced ATP production, mtDNA damage, elevated ROS production, and mitochondrial calcium overload. The pathogenesis of DOX-induced cardiomyopathy may involve dysregulated mitochondrial biogenesis and accelerated mitochondrial fragmentation (Songbo et al., 2019).

In murine cardiomyocytes, DOX has been demonstrated to induce mitochondrial fragmentation (Riba et al., 2017). In cardiomyocytes, DOX administration reduces the levels of fusion proteins, including Mfn1, Mfn2, and OPA1, shifting the mitochondrial fission/fusion balance toward fission (Tang et al., 2017; Prathumsap et al., 2022). This DOX-induced mitochondrial fission is not primarily linked to cell division or apoptosis but is associated with mitophagy, suggesting it may represent an adaptive response to DOX-induced stress (Songbo et al., 2019).

Recent research has increasingly focused on mitochondrial quality control systems as potential therapeutic targets to mitigate DOX-induced cardiotoxicity. Mdivi-1 alleviates DOX-induced cardiotoxicity by inhibiting Drp1 phosphorylation and mitochondrial fission (Gharanei et al., 2013). It also attenuates DOX-induced overactivation of PINK1/Parkin-mediated mitophagy, potentially contributing to the preservation of mitochondrial mass and function (Yin et al., 2018). Furthermore, cyclosporine A contributes to the maintenance of mitochondrial fusion by upregulating Mfn2 and OPA1 (Marechal et al., 2011). Additionally, melatonin and metformin, both widely used clinically, can enhance mitochondrial biogenesis by preserving mitochondrial function and homeostasis (Arinno et al., 2021). Furthermore, the function of the novel MTFP1 in doxorubicin (DOX)-induced cardiotoxicity remains unclear. However, studies have demonstrated that knockdown of Mtfp1 can reduce cardiomyocyte loss associated with DOX-induced cardiotoxicity. Consequently, modulating MTFP1 expression may represent a novel therapeutic strategy for managing chemotherapy-induced cardiotoxicity (Aung et al., 2017). Table 3 provides a summary of the role of mitochondrial quality control drugs in cardiovascular disease models.

**TABLE 3:** Summary of mitochondrial quality control-targeting drugs in cardiovascular disease models.

Disease	Drugs/Compounds	Effect
Sepsis	mdivi-1	Preserve mitochondrial function and mitigate apoptosis
Myocardial ischemia/reperfusion injury	mdivi-1	Enhances cell survival and delays mPTP opening
	P110	Reduces infarct size and adverse remodeling (myocardial I/R)
Diabetic cardiomyopathy	brain natriuretic peptide (BNP)	Protects against DCM by promoting OPA1-mediated fusion
Doxorubicin-induced myocardial apoptosis	mdivi-1	Alleviates cardiotoxicity by inhibiting Drp1-mediated fission and attenuating aberrant PINK1/Parkin-mitophagy
	ciclosporin A	Upregulates Mfn2 and OPA1, promoting fusion
	melatonin and metformin	Enhance mitochondrial biogenesis and function

## 5 Conclusion

Mitochondrial dynamics, governed by proteins such as Drp1, Mfn1/2, and OPA1, are essential for maintaining cardiac homeostasis by regulating energy production, quality control, and cell survival. Disruption of the fission/fusion balance is a key pathogenic mechanism in various myocardial diseases. In sepsis, decreased Mfn2 and increased Drp1 levels lead to mitochondrial fragmentation and dysfunction. During I/R injury, excessive Drp1-dependent fission exacerbates damage, whereas OPA1 overexpression or Drp1 inhibition exerts protective effects, albeit with potential impairment of basal mitophagy. Diabetic cardiomyopathy is marked by reduced expression of fusion proteins (Mfn1, Mfn2, OPA1), increased fission, and overproduction of reactive oxygen species (ROS), which contribute to insulin resistance and cardiac dysfunction. Similarly, doxorubicin-induced cardiotoxicity features downregulation of fusion proteins and increased fission, associated with dysregulated mitophagy. Targeting specific dynamin-related proteins, such as with Drp1 inhibitors (mdivi-1, P110) or strategies to enhance OPA1 or Mfn2, has demonstrated considerable therapeutic potential in preclinical models. However, the functional redundancy and multifaceted roles of these regulators—especially their involvement in critical processes such as mitophagy, apoptosis, and development—underscore the complexity of such interventions. Despite significant advances, several controversies and limitations remain. The dual—sometimes opposing—roles of dynamics proteins in fusion, fission, mitophagy, and apoptosis complicate therapeutic targeting. Many studies rely on genetically modified mouse models, which may not fully recapitulate human disease pathophysiology. Furthermore, tissue-specific and temporal regulation of these proteins is still poorly understood. Future research should prioritize elucidating the tissue-specific regulation and temporal dynamics of these processes during disease progression. Additionally, developing strategies to precisely modulate these pathways to restore mitochondrial and cardiac function, without compromising essential cellular activities, is crucial.
